# Development and pilot of an interprofessional pediatric resuscitation program for non-acute care inpatient providers

**DOI:** 10.1080/10872981.2019.1581521

**Published:** 2019-02-27

**Authors:** Ronish Gupta, Colleen Fitzgibbons, Christa Ramsay, Lindsey Vanderheiden, Christina Toppozini, Anna-Theresa Lobos

**Affiliations:** a Department of Pediatrics, Children’s Hospital of Eastern Ontario, Ottawa, ON, Canada; b Fellow, School of Education, Johns Hopkins University, Baltimore, MD, USA; c Department of Pediatrics, McMaster University, Hamilton, ON, Canada; d Children's Hospital of Eastern Ontario, Ottawa, ON, Canada

**Keywords:** Interprofessional education, resuscitation, simulation, teamwork

## Abstract

Multiprofessional ward healthcare providers are generally unprepared to assemble and engage in the initial resuscitation of pediatric inpatients. This is important as the performance of these first-responders, in the several minutes prior to the arrival of acute care support, may have significant effects on overall patient outcome. Accordingly, we aimed to develop and pilot a training program intended for non-acute care inpatient providers, relevant to their working context. Using the latest theory and evidence in medical education, we created an interprofessional, entirely in-situ, simulation-based small-group activity. The activity was then piloted for four months with the goals of assessing perceived usefulness, as well as implementation factors such as participant accessibility and overall resource requirements. A total of 37 interprofessional (physician and nursing) staff were trained in 16 small group sessions over four months. Post-participation questionnaires revealed that the activity was perceived to be highly useful for their practice; especially the rapid cycle deliberate practice instructional method, and the increased focus on crisis resource management. Resource requirements were comparable to, and perhaps less than, existing acute care training programs. This project describes the preliminary steps taken in creating a curriculum intended to improve interprofessional resuscitation performance across an institution.

## Background

Pediatric inpatient cardiopulmonary arrests are uncommon, but have significant health implications. Despite some improvements over time, a recent multi-center review of pediatric inpatient arrest events showed that rates of survival to hospital discharge were below 50% [1]. In response, code blue and medical emergency teams have been implemented to improve the delivery of acute care expertise to patients outside critical-care areas [2]. However, studies in both adult and pediatric populations show that prior to the arrival of acute care teams, initial resuscitative efforts by ward first-responders are variable and often sub-standard [3–5], potentially impacting patient outcome.

With infrequent clinical exposure to deteriorating or acutely ill patients, there are limited relevant and accessible ways for pediatric ward providers to maintain basic acute care skills. While there are structured educational opportunities provided by the American Heart Association, these courses [6] are generally too basic (e.g. Basic Life Support [BLS]) or too advanced (e.g. Pediatric Advanced Life Support [PALS]) for individuals whom either mainly or entirely work in non-acute patient care areas. Newer courses designed for non-acute care pediatric providers have been developed, such as Pediatric Advanced Emergency Assessment, Recognition, and Stabilization (PEARS), but remain challenging to feasibly administer regularly to large numbers of professional staff. Recently in California, medical educators recognized this gap and developed an interprofessional training program utilizing in-situ simulation scenarios and found improvements in participant self-confidence ratings [7]. This experience provides some initial strategies and may be expanded upon by several recent advances in resuscitation education science and best practice suggestions [8].

Building on the existing literature, our goal was to develop an updated simulation-based, context-specific, interprofessional training program specifically for ward-based pediatric providers. We also aimed to pilot the activity for perceived usefulness, as well as to understand potential implementation obstacles such as participant accessibility and resource requirements.

## Methods

### Setting and participants

The program was developed at a stand-alone children’s hospital. The facility is part of an academic health sciences centre, with 166 inpatient beds, and approximately 6000 admissions per year. Outside the acute care units, patients are cared for by over 60 residents and fellows, 200 staff physicians, 30 respiratory therapists, and 500 nurses. Pediatric residents participate in acute care training and simulation during their residency curriculum. The remaining providers, however, have no regular acute-care curriculum nor training requirements. Ward-based simulated mock codes, similar to those described by van Schaik et al. [7]. are held monthly with only a small proportion of ward providers able to participate.

### Program development

An organizing group of interprofessional staff (see author list) undertook the six-steps of curriculum development [9]. Following the aforementioned problem identification and general needs assessment, a targeted needs assessment was conducted locally. The institutional Emergency Response and Resuscitation Committee, whom reviews all inpatient cardiorespiratory arrests, was consulted to provide a list of common issues. Identified concerns included: delays in performing basic maneuvers (e.g. pulse-check), improper use of equipment (e.g. bag-valve mask), and a lack of establishing team leadership. Program learning objectives were generated based on this targeted assessment (Table 1).

**Table 1.** Resuscitation program for pediatric non-acute care providers learning objectives.

By the end of the session, participants will be able to…	
Cognitive	… list to the instructor, in the correct order, the five critical actions to perform following an in-patient code blue activation … describe to the instructor, specifically, the location of resuscitation equipment on the local unit
Psychomotor	… demonstrate the correct assembly and use of a standard ward self-inflating bag-valve mask system on an infant mannequin … demonstrate the correct assembly and use of standard ward suction equipment on an infant mannequin … demonstrate the correct technique for performing a pulse-check and high-quality chest compressions with a backboard on an infant mannequin
Affective/ Attitudinal	… demonstrate the value of having established leadership during a code blue event, by consistently identifying the leader, or assuming the leadership position as the first step in a simulated code blue scenario … collaborate with team members in a manner that ensures the five critical steps are consistently performed in the first five minutes of a simulated code blue scenario

A concept map was created to assist learners in attaining knowledge and process objectives (see Appendix 1). The concept map resembles standard pediatric life-support algorithms but focuses on the management steps expected of and available to ward providers at the institution, prior to the arrival of mobile acute care teams. Crisis resource management (CRM) skills, specifically leadership, were included and emphasized as previous studies have highlighted their importance to goal-oriented team functioning [10]. In contrast to acute care environments, the ward presents unique challenges as entirely ad-hoc teams of personnel must instantaneously assemble and function with virtually no notice or preparation.

The educational literature was then reviewed for pertinent instructional methods. Appropriate planning is essential for successful interprofessional educational activities [11]. Employing concepts from adult learning theory, we ensured the setting was entirely in-situ (in the clinical environment) and used equipment identical to those found on the wards. Instructors were also taught to prompt reflective dialogue with participants. In addition, we planned to track implementation related factors to help identify potential obstacles to wider-scale feasibility, sustainability, and accessibility, understanding that many educational innovations may be efficacious in research settings but are often practically limited by resource requirements.

From a simulation perspective, the program objectives required training individuals to perform specific critical tasks competently (e.g. pulse-check), as opposed to engaging in abstract thinking or problem-solving. Accordingly, we facilitated the sessions using Rapid Cycle Deliberate Practice (RCDP), whereby performance is directly observed, and learners are provided immediate feedback with the opportunity to repeat until mastery is achieved. RCDP has become a central element in resuscitation learning [8], and has shown some effectiveness specifically in the pediatric acute care context [12].

Next, guided by the implementation principles of the resuscitation education science literature [8], we prioritized making the program accessible to all ward providers in brief, small group sessions. Based on feedback from stakeholder groups, we concluded that a maximum participant activity duration of 50-minutes would balance effectiveness with resource investment.

### Implementation

The program was run by two instructors, beginning February 2017, with one instructor being experienced in mannequin transport and setup on the ward. Individual one-hour sessions consisted of 45-minutes of instructional time with an additional 5-minutes of built-in feedback and evaluation time, and 10-minutes of instructor cleanup and setup (see Appendix 2 for detailed activity outline). In general, three to four sessions were run consecutively, thus typically requiring a half-day time commitment from instructors.

Program participants were ward physicians (attending physicians and trainees) and ward nurses. At least one physician and one nurse were required per group, with a goal of three to four participants total. Physicians scheduled their session ahead of time, while ward nurses on-shift were invited to participate. Of the 45-minutes of instructional time, instructors spent the first five minutes performing introductions and reviewing the program objectives and rationale. The next 15 minutes were spent demonstrating and then observing participants perform technical skills by retrieving and using equipment identical to those found on the ward. Participants were coached until each skill was done correctly. The remaining 20–25 minutes was spent running RCDP arrest simulations with the entire group (see Appendix 3 for an example RCDP scenario). Importantly, participants were not permitted to select a leader ahead of each simulation; as in real life, leadership was to be negotiated and established dynamically during the event. With each simulation, instructors observed and provided direct feedback to participants for both medical and teamwork skills.

### Data collection

Participants completed anonymous program evaluation forms at the end of each session. In addition to basic demographic information, the forms utilized multiple-choice questions, likert scales, and comment spaces to collect information on perceived usefulness and potential areas of improvement.

### Data analysis

Quantitative data from evaluation forms were combined and summarized descriptively. Valid percentages were calculated when required. Comments were analyzed directly by identifying key words and phrases, and then clustering associated terms into themes.

### Permissions

Program participants were not identified as part of this analysis, which was conducted under a quality improvement designation from the institutional research ethics board.

## Results

A total of 21 sessions were scheduled on seven days from February to May 2017. Five sessions were cancelled due to insufficient attendance, resulting in 16 completed sessions (76%). In eight sessions (50%), a ward nurse was not available, and a ward nurse educator participated instead. A total of 37 individuals participated in the pilot sessions, and 31 evaluation forms were submitted for a completion rate of 84%. Respondents were: ward nurses (6, 19%); residents (6, 19%); staff general pediatricians (5, 16%); and staff subspecialty physicians (14, 45%).

Most participants indicated that all parts of the program were either ‘very useful’ (should be kept in the program), or ‘extremely useful’ (definitely keep in the program) (Table 2). Participant written comments were almost entirely positive and several common themes emerged (Table 3). These included: use of in-situ location and equipment, opportunity to practice crisis management with interprofessional colleagues, and rapid-cycle simulation training. Program suggestions included: nurses not requiring a review of suction equipment; and requests for longer or follow-up sessions.

**Table 2.** Program pilot participant ratings of usefulness of each element of the training program.

	Degree of Usefulness (recommendation to remove from or keep as part of activity)				
	Not at all (definitely remove)	Slightly (consider removing)	Somewhat (keep if time permits)	Very (should keep)	Extremely (definitely keep)
Introduction and rationale	0	0	0	8 (26%)	23 (74%)
Review of location, assembly, and use of bag-valve mask	0	0	1 (3%)	5 (16%)	25 (81%)
Review of location, assembly, and use of suction equipment	0	1 (3%)	0	6 (19%)	24 (77%)
Demonstration of algorithm by instructors	0	0	1 (3%)	3 (10%)	25 (86%)
Participant-led simulations	0	0	0	2 (7%)	28 (93%)

**Table 3.** Themes from program pilot participant written comments with example quotes.

Program Areas of Strength	Use of in-situ location and equipment for training *‘This was essential we often don’t know where the equipment is located’* *‘It’s extremely useful to have a situation in the room- situational awareness knowing how to use the equipment that is actually on the ward.’*
	Opportunity to practice crisis management with interprofessional colleagues *‘Loved this! Help[ed] bring down some of the anxiety and allow[ed] us to practice taking a step back to gain control’* *‘This was great! Love that it is interdisciplinary’*
	Process of rapid cycle deliberate practice training *‘Enjoyed starting and stopping scenario with suggestions for improvement’* *‘Great to have feedback immediately would recommend to all [ward providers]’* *‘Very helpful to be able to practice’*
Program Suggestions for Change	Nurses not requiring review of suction equipment *‘Not necessary for nursing staff who use this equipment on a daily basis’*
	Request for longer or follow-up sessions *‘More practice welcome’* *‘[W]ould have liked longer (maybe 2 sessions)’* *‘Should have this done at regular interval[s]’*

## Discussion

Cardiorespiratory arrest is uncommon in pediatric inpatients [1], and pediatric ward providers are generally unprepared to manage these situations [3]. Thus, an educational opportunity exists to train providers on resuscitation techniques in the minutes prior to the arrival of acute care teams. In this study, we used rigorous curriculum development methods as well as the latest educational theory and evidence to design a training activity targeting this gap. The activity was trialed on a pilot basis, with the goals of examining its perceived usefulness, and understanding the potential barriers and obstacles to wider-scale implementation.

Existing literature has shown improvements in individual and team performance using simulation and the RCDP technique [12], and is recommended by resuscitation education literature [8]. Our goal was to harness this method and translate it into an effective interprofessional activity that could be introduced with the minimum amount of human, equipment, and time resources possible. This was to maintain the potential for sustainability, an important limiting factor for many healthcare programmatic interventions [13].

From an effectiveness standpoint, the program was well received by participants. Most exhibited high degrees of self-motivation as they recognized the relevance of the content and skills being taught to their direct patient care environment. Practicing CRM skills through ad-hoc interprofessional team formation was also perceived as valuable.

From a resource perspective, the activity appears to require similar and possibly fewer human and equipment resources compared to existing simulation acute care training activities. No specialized simulation space is required aside from an empty existing ward room, and a single low-technology mannequin is adequate. Compared to existing programs, and presuming full attendance, two instructors each spending 45-hours per year would be able to train up to 36 learners in PALS (12 participants per session, 3 sessions), up to 60 learners in PEARS (12 participants per session, 5 sessions), and up to 180 learners in our program (4 participants per session, 45 sessions). It should be acknowledged that these numbers simply reflect the reach to a learner audience, and not necessarily equivalence in training or scope of practice. Forty-five hours of individual instructor time per year equates to approximately 4-hours (or one half-day) of simulation work per month. Feedback from program instructors suggested the investment was reasonable given their existing clinical and academic commitments, especially since sessions required little to no extra pre- or post-work. Finally, although the scale of our pilot phase would be inadequate to train large numbers (e.g. several hundreds) of staff, including additional sessions and instructors could potentially accommodate larger learner populations.

Considering accessibility, participants considered the short, 50-minute sessions on the inpatient units to be desirable and more easily accessed than standard pediatric resuscitation courses. However, there were identified challenges with accessibility, notably with ward nurse recruitment. The plan for ad-hoc on-shift ward nurse participant recruitment led to several cancellations. It seems that in order to maintain consistent ward nurse participation, deliberate plans for duty coverage and/or protected time are required on planned training dates. Alternatively, training dates may be scheduled to coincide with nurse orientation and education dates. In either case, given that ward nurse participation is a vital part of the interprofessional activity, seeking involvement as well as support from nurse managers and administrators at the outset is recommended.

Overall, we developed and piloted a targeted and specific interprofessional resuscitation training activity for pediatric ward providers, that participants perceived to be highly useful. Resource requirements are not insignificant, but are comparable to or less than existing acute care training activities. Future steps include improving the capacity for participant attendance and measuring the program’s impact on learning, behavior, retention, and patient outcomes.

## Appendices Appendix 2: ‘First Five Minutes’ Session Outline

**Table:**

Individual Session Structure
Minute: 00:00–05:00
● Introductions of participants and instructors● Review of program objectives and rationale based on needs assessment data● Group review of ‘First Five Minutes’ algorithm and concept map (Appendix 1)
Minute: 05:00–20:00
● Instructors demonstrate on a mannequin the following skills using equipment identical to those found on the ward: ○ Chin-lift, jaw-thrust, and airway suctioning ○ Bag-valve mask ○ Pulse-check and chest compressions with backboard ○ Pupil and level of consciousness assessment ● Instructors directly observe each participant’s performance of each skill above, with feedback and coaching as necessary until performed correctly
Minute: 20:00–45:00
● Rapid Cycle Deliberate Practice (RCDP) scenarios (Appendix 3) ○ Several scenarios are run, each 3–4 minutes in duration with approximately 1 minute of instructor feedback ○ Scenarios are similar, and consist of an instructor playing the role of a confederate nurse or parent activating the code blue system for a child ○ For each scenario, participants are randomly assigned in order of arrival to the room, and occasionally in groups, to simulate a typical ward response ○ Leadership of each scenario is to be established, and negotiated by participants dynamically
Minute: 45:00–50:00
● Protected time for participants to complete anonymous evaluation forms
Minute: 50:00–60:00
● Instructor time for clean-up and preparation for next group

## Appendices Appendix 3: ‘First Five Minutes’ Example Rapid Cycle Deliberate Practice Scenario

The scenario should represent cases typical or representative of those found on the ward. Excessive details are generally unnecessary as the scenarios are extremely short in duration, and meant to have participants practice establishing leadership amongst the group, as well as performing initial life support measures in the ward environment, and not necessarily engaging in advanced analyses of each given case.

*The following is an example*:

**Table:**

**Preparation**	
● Infant mannequin (low technology), not on monitors● 1^st^ instructor acting as a parent confederate, 2^nd^ observing instructor in room● Ward crib● Wall suction and accessories● Self-inflating bag-valve mask system packaged in room/ward in standard fashion● Crash cart containing chest compression backboard stationed outside room● Typical ward light source for pupil exam● Available monitoring leads, probe, and blood pressure cuff	
**Scenario**	
*Participants all wait outside the ward room and have either self-assigned or instructor-assigned order/timing of room entry after code blue activation. The order/timing of entry should change with each scenario.*	
Time: 00:00 – 00:15	Confederate activates code blue/yells for help within room. First responding participant is given brief stem:● ‘I’ve just found my baby completely limp and pale, I don’t think she’s breathing! She is 5-months-old, admitted last night for bronchiolitis, she seemed fine when I finished feeding her just a few minutes ago’
Time: 00:15 – 00:30	Initial assessment reveals no respiratory effort, and no pulse.
Time: 00:30 – 03:30	Initial responding participant, and subsequently arriving ones work as a team to perform the First Five Minutes algorithm together● Establish leadership structure initially and dynamically ongoing● Position and suction airway as needed● Initiate BVM ventilation initially with room air, eventually 100% FiO_2_● Initiate chest compressions with back-board, 15:2 ratio with ventilation● Pupil and AVPU status● Place monitors to obtain full vital signs● Mention obtaining blood glucose, preparing IV/IO access
Time: 03:30 – 04:00	Scenario leader provides brief SBAR handover to observing instructor
Time: 04:00 – 05:00	Short debrief and feedback between participants and instructors
